# Interfacial Adhesion of Mouthrinses to Orthodontic Metal Wires: Surface Film Viscoelasticity Effect

**DOI:** 10.3390/ma18174065

**Published:** 2025-08-29

**Authors:** Stanisław Pogorzelski, Krzysztof Dorywalski, Katarzyna Boniewicz-Szmyt, Paweł Rochowski

**Affiliations:** 1 Institute of Experimental Physics, Faculty of Mathematics, Physics and Informatics, University of Gdańsk, Wita Stwosza 57, 80-308 Gdańsk, Poland; stanislaw.pogorzelski@ug.edu.pl (S.P.); krzysztof.dorywalski@ug.edu.pl (K.D.); 2 Department of Physics, Gdynia Maritime University, Morska 81-87, 81-225 Gdynia, Poland; k.boniewicz@wm.umg.edu.pl

**Keywords:** polymer–metal prosthesis, dental wires, mouthrinse spreading, surface adhesion, wettability energetics, surface energy, spreading dynamics

## Abstract

This study concerns the evaluation of adhesive and wettability energetic signatures of a model orthodontic wire exposed to commercial mouthrinses. The surface wetting properties were evaluated from the contact angle hysteresis (*CAH*) approach applied to dynamic contact angle data derived from the original drop on a vertical filament method. Young, advancing, receding *CA* apart from adhesive film pressure, surface energy, work of adhesion, etc. were chosen as interfacial interaction indicators, allowing for the optimal concentration and placement of the key component(s) accumulation to be predicted for effective antibacterial activity to eliminate plaque formation on the prosthetic materials. Surfactant compounds when adsorb at interfaces confer rheological properties to the surfaces, leading to surface relaxation, which depends on the timescale of the deformation. The surface dilatational complex modulus *E*, with compression elasticity *E_d_* and viscosity *E_i_* parts, determined in the stress–relaxation Langmuir trough measurements, exhibited the viscoelastic surface film behavior with the relaxation times (0.41–3.13 s), pointing to the vertically segregated film structure as distinct, stratified layers with the most insoluble compound on the system top (as indicated with the 2D polymer film scaling theory exponent y = 12.9–15.5). Kinetic rheology parameters could affect the wettability, adhesion, and spreading characteristics of mouthrinse liquids.

## 1. Introduction

Chrome-nickel stainless steel orthodontic wire is used in complex dental structures for the production of clasp wire, sublingual arches, and pressed palatal plates apart from and accompanied with polymeric (largely PMMA) parts. Therefore, it is interesting to evaluate the specific adsorptive, adhesive, and wettability characteristics of metallic substrates in relation to polymeric ones that have been studied elsewhere [1,2].

The surface rheology kinetics parameters of mouthrinse fluids can significantly influence the antibacterial effectiveness of the oral care product. This is important when creating the composition of a medical product to ensure that the active ingredient accumulates in the areas where it is expected to be effective, i.e., on the interfacial surfaces or in the solution volume. This problem was already addressed previously—see Figure 1 of [2].

Thus far, the research has focused on the adsorption and surface wettability parameters for mouthrinses and denture cleansers in contact with a polymer commonly used in the production of dentures (PMMA) under assumption of a steady-state liquid coating process. The analysis was performed on measured static liquid–solid contact angles and equilibrium rheological surface properties [1,2].

However, the surface rheology kinetics parameters (the complex surface dilatational modulus, for instance [3]) can affect efficiency of interfacial interactions between mouthrinse and a solid surface, which depends on the timescale of the surface deformation in reference to the characteristic relaxation times of processes occurring at the air–liquid interface, further mediating interfacial vertical molecular segregation or architecture of liquid-composing compounds [4,5].

In our multicomponent liquid surface rheology studies, miscibility of film compounds and film structure evolution could be expressed by the scaling exponent *y* by adopting the 2D polymer film scaling theory [4]. The highest *y* values point to the vertically segregated film structure forming nearly separate layers at the interface. Moreover, the stress–relaxation measurements revealed relaxation processes at the interface with characteristic times *τ_i_* (*i* = 1, 2…), suggesting the presence of diffusion-controlled and structural organization relaxation phenomena [3,5].

The aim of the study was twofold: (1) to determine the basic adhesive properties in the orthodontic metal wire to a commercially available mouthrinse system using Chibowski’s formalism based on the hysteresis of contact angles (*CAH*), as measured by the original method (drop on a vertical filament [6,7]), and (2) to estimate the role of surface viscoelasticity of the fluids and the time scale of relaxation processes exhibited in the process of intensive movement of the cleaning fluid during the application process. In particular, the viscoelastic properties (complex surface dilatational elasticity modulus) of the surface film of mouthrinses influence the effective surface tension (*γ_eff_* ↓) of the spreading liquid and dynamic contact angle hysteresis (*CAH* ↑) with the solid substratum further mediating the apparent adhesion–wettability signatures (work of adhesion ↓) and penetration coefficient (*PC* ↓) of the solid–liquid interfacial system.

## 2. Theoretical Background

### 2.1. Interfacial Adsorptive and Wettability Characteristics

In order to quantify the adsorptive and wettability effect of commercially available mouthrinses on the stainless-steel orthodontic wire, Gibbs excess *Γ*, surface activity, partitioning coefficient, critical micellar concentration *CMC*, and surface entropy *S_s_* were derived from the surface tension–concentration *γ_LV_(c)* and the surface tension—temperature *γ_LV_ (T)* dependences, which were already introduced in [5]. Surface wettability of the model system was characterized from three measurable quantities: liquid surface tension and two dynamic contact angles—advancing (*θ_A_*) and receding (*θ_R_*) contact angles—analyzed with the contact angle hysteresis (*CAH = θ_A_ − θ_R_*) Chibowski’s approach. In particular, Young’s equilibrium contact angle *θ_Y_*, adhesive layer surface pressure *Π*, work of adhesion *W_A_* and cohesion *W_C_*, surface free energy *γ_SV_* including its polar and dispersive components, adhesional tension *γ_LV_ cos θ_A_*, and surface excess ratio *a = Γ_SL_/Γ_LV_* (which were specified in detail in [5]) were selected as interaction strength indicators at interfaces. Moreover, the chosen parameters are designed to characterize the particular interfacial regions in the vapor–liquid–solid system, as pointed to in Figure 1 of [2].

### 2.2. Interfacial Miscibility of Surface-Active Compounds

The polymer film scaling theory can be used to analyze the spatial distribution of surfactants found in multicomponent natural films [8].

The 2D polymer film scaling theory was adopted here by measuring the high-frequency limit of the surface elasticity Gibb’s modulus, *E_isoth_*, and applied to a multicomponent surfactant mixture in order to derive the scaling exponent *y* being a molecular miscibility spatial distribution indicator [4]. As depicted schematically in Figure 1 of [4], an increase in *y* from ~3.5 through ~8 and finally to over 10 (representing ‘good’ and ‘poor’ solvent behavior) is likely to be correlated with a transition in the molecular architecture, beginning with a homogeneous monolayer and progressing through a heterogeneous film with surface-active components that are segregated into patches, surface micelles, or domains, and finally forming a vertically layered structure with the most insoluble (and most surface-active) compound at the top.

The parameter *y* can be determined from the *(Π -A)_T_* isotherm, i.e., from the slope of the initial, straight-line part of the *E_isoth_(Π)* relationship in the range of low surface pressures (0 < *Π* < 2–3 mN m^−1^) using the relationship [4]:

(1) $$E_{isoth}=y\Pi$$

The condition for using the relationship is the registration of isotherms corresponding to the conditions of “effective insolubility” of the film material (at a high surface film area *A* compression rate of the interfacial region), i.e., when *t_obs_ << τ*—relaxation times of transient processes in the film. The time of observation *t_obs_* is defined as [9]:

(2) $$t_{obs}={\left(\frac{\Delta A}{A_{0}\Delta t}\right)}^{-1}$$

where *A*_0_ is the initial film area, and Δ*t* is the surface deformation time. In fact, the effect depends on the value of a dimensionless parameter—the so-called Deborah number (De), defined as the ratio of the characteristic time *τ* of the relaxation process in the film to the observation time [9]:

(3) $$De=\tau /t_{obs}$$

### 2.3. Interfacial Relaxation Processes-Dilatational Viscoelasticity Modulus

The modulus of expansion of surface elasticity *E* is generally a complex quantity when relaxation processes of the interfacial system are revealed at the molecular level, together with the introduced deformation of the surface volume, and related to the reorientation or diffusion exchange of molecules therein and with the subsurface region. Any relaxation process in films leads to dilatational viscoelasticity. The surface dilatational viscoelastic modulus *E* is a complex quantity composed of real *E_d_* (dilatational elasticity) and imaginary *E_i_* (=*ωη_d_*, where *η_d_* is the dilatational viscosity) parts, *E = E_d_ + iE_i_ = E_0_ cos φ + iE_0_ sin φ*; *E_0_=* − Δ*Π/(*Δ*A/A)*, where *φ* is the loss angle (with *tan φ = E_i_/E_d_*), and ω is the angular frequency of periodic area oscillations [3]. First, the surface pressure–time (*Π* − *t*) response of a film to a step-wise, rapid (Δ*t* < 1.5 s) relative surface area deformation Δ*A/A_0_* (= 0.07–0.23) is registered in order to derive the main relaxation times (*τ*_1_*, τ*_2_…) by plotting ln *Π(t)*, and then the complex modulus parts were determined, as demonstrated in [3]. At sufficiently low film area compression rates (*De* << 1), the dilatational viscoelasticity modulus *|E|* can be approximated by *E_isoth_* derived from the surface isotherm:

(4a) $$\left|E\right|\sim E_{isoth}=-A{\left(\frac{d\pi }{dA}\right)}_{T}$$

with:

(4b) $$\left|E\right|=\sqrt{E_{d}^{2}+E_{i}^{2}}$$

Any deformation of the surface covered with a two-dimensional viscoelastic film with modulus *|E|* will be met by an opposing reaction in the form of a corresponding change in surface tension Δ*γ = |E| (*Δ*A/A*_0_*)* depending on the surface elasticity and the relative magnitude of its change [10]. The effective surface tension of the surfactant solution *γ_eff_* has the value:

(5) $$\gamma_{eff}=\gamma_{LV}-\left|E\right|(\Delta A/A_{0})$$

The rate of change of the effective surface tension in time is described by the relationship (at constant *γ_LV_*):

(6) $$\frac{d\gamma_{eff}}{dt}=-\left|E\right|\frac{d(\Delta A/A_{0})}{dt}$$

## 3. Materials and Methods

### 3.1. Dynamic Contact Angle Determination

Stainless steel, trade name Remanium (Cr Ni) 0.8 mm diameter orthodontic wires (Dentaurum GmbH & Co. KG, Ispringen, Germany), were selected as a model substratum. The principal physical properties of five commercially available mouthrinses, namely, density *ρ*, viscosity *μ*, surface tension *γ_LV_*, and acidity (pH), were already evaluated, as shown in [1], and are collected in Table 1 therein.

**Figure 1:**
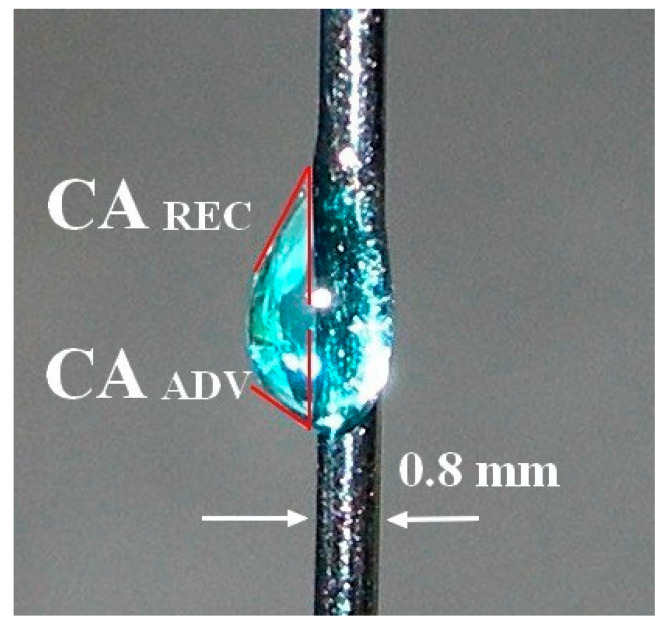
Dynamic contact angles determination from exemplary mouthrinse drop at metallic wire; *γ_LV_* = 39.9 mN m^−1^, *θ_A_* ($\equiv CA_{ADV}$) = 39.3°, *θ_R_* ($\equiv CA_{REC}$) = 26.8°, *CAH* = 12.5° at *T* = 22 °C. The experimental data revealed that CAH increases with an increase in the filament radius. Particularly, the advancing CA was more strongly affected than the receding one.

The experimental setup and dynamic *CA* determination methodology are presented in Figure 1 of [6] and described in detail therein. A filament-wire measuring 20–25 cm long was partially enclosed by the cell saturated with the vapor of the test liquid (mouthrinses and water as a reference). In brief, due to gravity action, the droplet started to move down to the cell, leaving a portion of liquid in a thick wetting film on the filament behind from the droplet. When the weight of the moving droplet decreased enough to fulfill the condition of mechanical equilibrium, the droplet stopped as originally argued in [7]. The analysis of drop shape at the moment of its fixation gives the (both advancing and receding) *CA* at given radius of the filament and corresponds to the maximal hysteresis *CAH*. An exemplary image of Ortho Salvia Dental drop on vertical metallic wire filament is depicted in Figure 1.

The classic hydrodynamic model of the phenomenon of liquid spreading on a solid surface and the profile of the interphase surface points to the conditions required for the kinematic parameters (so-called dimensionless flow numbers), which allow reliable and repeatable *CA* to be achieved [11]. In particular, the ratio of viscosity forces to capillary forces is quantified in terms of the capillary number *Ca*:

(7) $$Ca=u\frac{\mu }{\gamma_{LV}\;}$$

where *u* is the liquid–solid contact line front speed, and μ is the liquid viscosity [12]. Very low values of *Ca* (10^−3^ to 10^−5^) correspond to a thermodynamic equilibrium at the liquid–vapor interphase, and the sessile drop shape can be approximated with a spherical cap. As a consequence, the dynamic contact angle *θ_d_* instead of the Young *θ_eq_*, equilibrium contact angle is observed, which depends on *Ca*, and can be expressed by the empirical dependence [13]:

(8) $$cos\theta_{d}=cos\theta_{eq}-2\left(1+\cos\theta_{eq}\right)Ca^{0.5}$$

It is assumed that the liquid–solid contact line shape is properly formed when the surface tension forces prevail over the viscosity forces (*Ca* << 1) and the gravity force (*Bd* << 1), where *Bd* stands for the Bond number expressed by the following expression:

(9) $$Bd=\frac{\rho g\;D^{2}}{\gamma_{LV}}$$

where ρ stands for the liquid density, while *D* represents a characteristic dimension of the system (e.g., contact line length, droplet radius). Finally, the Weber number $We=\rho u^{2}D/\gamma_{lv}$ defines the ratio of inertia to capillary forces and needs to be <<1.

### 3.2. Surface Rheology: Static and Kinetic Signatures

To perform force–area *(Π-A)_T_* isotherm studies on mouthrinse air–liquid interfaces, the conventional Langmuir trough area *A*_0_ (=1200 cm^2^) was compressed with an average deformation speed of *v* = 0.6 cm^2^ s^−1^ by moving two PTFE sliders toward each other symmetrically around the film pressure sensor. Surface pressures were measured with a Wilhelmy plate technique using a piece of filter paper, as described in detail in [4].

Dynamic film characteristics were evaluated from the stress–relaxation studies [3], using a novel frame-shaped Langmuir trough apparatus (see Figure 2 in [3]). The surface pressure–time response *Π(t)* of a film to a rapid step (Δt = 0.19–1.1 s) relative surface area deformation Δ*A/A*_0_ (=0.07–0.23) applied to the sample by barrier movement was registered for several minutes. The reported structural and viscoelastic surface parameters stand for an average value over 6–9 measuring runs performed for the given sample.

## 4. Results and Discussion

### 4.1. Energetics of Wettability

In reference to Table 1, the value of the static Young, equilibrium contact angle *θ_Y_* (cos *θ_Y_* = ½ cos *θ_A_* + cos *θ_R_*) for the pure water–stainless steel reference system (=68.0°) is significantly higher than for the other probe liquids, varying in the range of 20.7° (Eurodont) to 33.6° (Ortho Salvia Dental); as such, they can be considered hydrophilic systems (θ < 90°). Generally, values of *γ_SV_* point to the low energy surface of the metallic substrata, ranging from 29.2 to 34.2 mJ m^−2^ if referring to the reference case (=42.2 mJ m^−2^). For all of the studied solutions, an increase in exposure time leads to the same trend,—*θ_Y_* ↓, *γ_SV_* ↑, and *W_A_* ↑—suggesting the transition processes occurring at the solid–liquid interface. There are further parameters collected in Table 1 that are suitable and sensitive enough for the quantitative evaluation of liquid–surface interaction strength. *CAH* values are apparently low, varying from ~12° (Dentalux + Alc.) to ~15° (Eurodont) in reference to the water case (=27.7°). The advancing (ranging from 26.7° to 39.3°) and receding (contained within 12.2–26.8°) contact angles reflect a surface activity diversity of the surface-active components forming a mouthrinse mixture. It is known that *θ_A_* and *θ_R_* correspond to the most hydrophobic and hydrophilic components, respectively. The adhesive layer 2D film pressure *Π*, which varies from 2.5 to 4.7 mN m^−1^, and values of *W_A_* (63.8–70.8 mJ m^−2^) are both much lower than those derived for the reference system (*Π* = 28.4 mN m^−1^; *W_A_* = 96.8 mJ m^−2^), which suggests that the mouthrinse solution is capable of coating the surfaces previously covered with less surface-active substances. Since *W_A_* = *γ_LV_* + *γ_LV_* cos *θ_A_*, the adhesional tension contribution to *W_A_* (as the ratio *γ_LV_* cos *θ_A_/W_A_*) varied within 0.43–0.47 and attained only 0.25 for pure water. It seems that such a surface wettability energetics parameter turns out to be the adhesive strength removal ability of mouthrinse fluids. One can note that *γ_SL_* = *γ_SV_* − *γ_LV_* cos *θ_A_*; thus, the second term is responsible for the surface free energy difference. For the total *γ_SV_* measured here, dispersive interactions ($\gamma_{SV}^{d}$ = 26.7–31.4 mJ m^−2^) dominate over the non-dispersive, i.e., polar ($\gamma_{SV}^{p}$ = 0.9–2.9 mJ m^−2^), components in agreement with the general dependence of surface energy components and bioadhesion on total *γ_SV_* [14]. Variability of the wettability parameters in relation to the efficiency of microbial biofouling at solid substrata is considered in detail in Section 4.4 of our paper [1].

The relative adsorption *a = Γ_SL_/Γ_SV_* can be derived from the *γ_LV_* cos *θ_A_* plotted versus *γ_LV_*, where the slope *a* = d(*γ_LV_* cos *θ_A_*)/d(*γ_LV_*), as shown in [1]. Generally, the absolute values of the *a* parameter were lower than 1, which suggests a lower surface concentration of the adhered material at the *S/L* than at the *L/V* interface. The excesses ratios derived using a linear best-fit procedure were all positive and equal to 0.77–0.89, which points to the hydrophilic adsorption interaction mechanism at the interface with complex multilayer vertically segregated molecular structures [2]. For instance, the conventional surfactants revealed negative slopes (−0.33 to 0.17), which means that they are adsorbed at the PMMA–solution surface via hydrophobic interactions [15].

**Figure 2:**
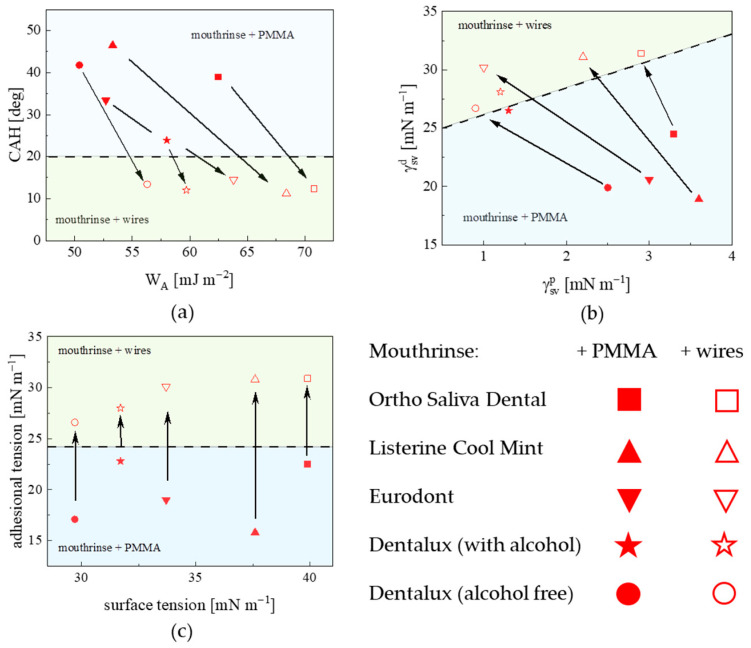
(**a**) Contact angle hysteresis (*CAH*) plotted versus work of adhesion (*W_A_*); (**b**) dispersive $\gamma_{SV}^{d}$ versus polar $\gamma_{SV}^{p}$ terms as a function of total $\gamma_{SV}$; (**c**) adhesional tension versus surface tension for the studied mouthrinses (at original concentrations) deposited on polymeric (PMMA) and metallic substrata at T = 22 °C. The border (dotted) line between data regions corresponds to these two interfacial systems. The selected parameter substratum-specific evolution, for the particular mouthrinse, is indicated with an arrow.

It is important to determine the differences in the adhesive properties of oral rinses to a polymer (PMMA) surface and those occurring next to metallic surfaces within the same integrated denture system. The different properties of the surface interaction of the mouthrinse fluids with two types of substrata, polymer (PMMA) and metallic, can be reflected in the distribution of experimental points on the 2D plane of the *CAH* dependence plotted as a function of *W_A_* (Figure 2a) and the relation between the dispersive $\gamma_{SV}^{d}$ and polar $\gamma_{SV}^{p}$ (Figure 2b) components of the total surface free energy. As presented in Figure 2a, the PMMA-corresponding region covered an area *CAH* of 23.9–46.5° and *W_A_* of 50.4–62.5 mJ m^−2^, which is apparently separated from the metallic one with *CAH* (11.2–14.5°) and *W_A_* (56.3–70.8 mJ m^−2^), for each of the oral rinses. From Figure 2b, it is evident that forces of a dispersive nature prevailing with the dispersive term $\gamma_{SV}^{d}$ contribution to the total free energy account for up to 92–97% for metallic surfaces and are lower (84–95%) for the polymeric-treated surfaces. The distribution of the experimental points in Figure 2b for each of the rinses allows one to distinguish two distant regions of coordinates ($\gamma_{SV}^{d}$, $\gamma_{SV}^{p}$) of (18.9–24.5, 1.3–3.6) for polymeric substrata and (26.7–31.4, 0.9–2.8) mJ m^−2^ corresponding to metallic ones. Data presented in Figure 2c allow the slope of the *γ_LV_* cos *θ_A_* vs. *γ_LV_* dependence, related to the surface surfactant excess ratio *Γ_SL_/Γ_SV_*, to be determined. Values of the slopes for the mouthrinse–metal system turned out to be positive, which indicates that the adsorption mechanism is based on hydrophilic interactions [15].

The results demonstrate that *CAH* originates from a single source, i.e., the difference in solid–liquid interaction in the vicinity of the contact line during advancing and receding contact line propagation modes. It is argued that large *CAH* is an indicator of stickiness, and adhesion is correlated to *θ_R_* only. To sum up, the solid substratum kind transition from the polymeric to metallic one, for the same mouthrinse, leads to the following adhesive–wettability parameters evolution: *θ_Y_* ↓, *θ_A_* ↓, *θ_R_* ↑, *CAH* ↓, *Π* ↓, adhesional tension ↑, $\gamma_{SV}\;$↑, $\gamma_{SV}^{d}$ ↑, $\gamma_{SV}^{p}$ ↓. The particular values of *CAH*, *W_A_*, and $\gamma_{SV}$ determined in CA studies can be useful in the selection of the most specific model liquids for further dental material wettability adjustment recommendations.

### 4.2. Metallic Wire Wettability vs. Mouthrinse Dissolution

The exemplary mouthrinse product (Ortho Salvia Dental) of original concentration (*C*_0_) was diluted with ultrapure water and then deposited at the metallic surface to obtain *CA* data. The wettability energetics parameters for the system are collected in Table 2. As a first step, from *γ_LV_(C/C*_0_*)* dependence, the concentration corresponding to saturation surface adsorption, so-called *CMC* (critical micellar concentration), was determined. They are already summarized in Table 2 of [1] for all the studied rinse fluids.

Similarly, like for several regular surfactant solutions, for *C > CMC* (here 0.03 *C*_0_), no noticeable variability in *CAs* and the related parameters is observed. Continuous increases in *θ_A_*, *CAH*, *Π*, *W_A_*, $\gamma_{SV}$, and $\gamma_{SV}^{p}$ were observed in the sub-micellar range (*C < CMC*), whereas the remaining quantities of *θ_R_* (23.8–25.4°), adhesional tension (28.4–33.1 mN m^−1^), and adhesional tension/*W_A_* ratio (0.39–0.31) just fluctuated around the mean values. The serial mouthrinse dissolution leads to the enrichment of the interfacial region with water polar molecules ($\gamma_{SV}^{d}$ ↑) forming more compact monolayers of higher 2D pressure (*Π* ↑). It should be noted that the excesses ratio (*a*) derived for pre-micellar and post-micellar concentration regions for the mouthrinse–polymeric system demonstrated negative values [1], which is evidence of the hydrophobic nature of the adsorption mechanism. However, as stated above, in the considered metallic–mouthrinse case, we are concerned with hydrophilic adsorption interactions. This could lead to different multilayered structure formations at interfaces as postulated in [2], where particular surface rheology and thermodynamics have to be considered. Studies reported in Section 4.3 and Section 4.4 revealed a complex molecular heterogeneous architecture of the *L/V* interfacial region of particular viscoelasticity and thermodynamics with the relaxation processes of differentiated origin and time scales.

### 4.3. Interfacial Molecular Architecture—Components Miscibility

**Figure 3:**
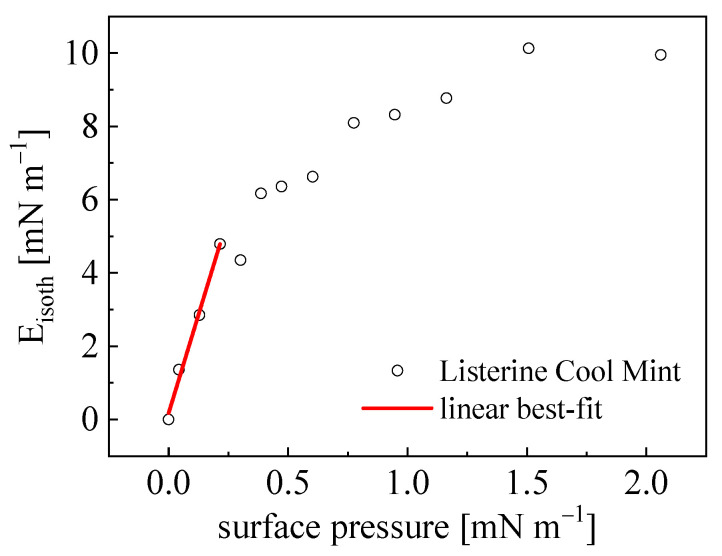
Dilatational elasticity modulus *E_isoth_* versus surface film pressure *π* derived from the surface isotherm *(Π -A)_T_* for Listerine Cool Mint solution at *C* = *C*_0_. Performed in a Langmuir trough at *T* = 22 °C, Δ*A*/Δ*t* = 28.3 cm^2^ s^−1^; *y* = 14.7 ± 0.3.

The local momentum due to the Marangoni effect (as a result of the surface deformation-induced surface stress Δ*γ_LV_*) can be quantified as the pseudo-surface diffusion coefficient $Du=E_{0}h/4\mu$ and leads to the limiting time $t_{lim}=L_{0}^{2}Du$; see Equations (6) and (7) in [4]. Here, *L*_0_ is the total length of the monolayer (Langmuir trough length = 35 cm), *h* is the liquid layer thickness (=0.7 cm), and μ and ρ are the liquid bulk viscosity and density (of water assumed). The quantity *E_0_* is the high-frequency limit of the surface modulus and can be derived from the (*Π -A*)_T_ isotherm dependence and identified with (*E*_0_~*E_isoth_*), as presented in Figure 3. For the considered exemplary system, *E*_0_ = 22.0 mN m^−1^, and *Du* = 370.9 cm^2^ s^−1^, which leads to $t_{lim}$ = 3.3 s. It was found that $t_{obs}$ >> $t_{lim}$, which fulfills the “effective” insolubility condition (according to the Equation (2), $t_{obs}$ = 42.4 s). Moreover, $t_{lim}$ values are of the order of the relaxation times *τ_i_* revealed in the stress–relaxation studies (Section 4.4). It can be noted that the isotherm studies performed on the same sample but with the slower area Δ*A/A*_0_ compression speed (=3.5 cm^2^ s^−1^ with the $t_{obs}$ = 344.8 s), corresponding to the system isotherm registration at its thermodynamic equilibrium, exhibited lower *E_isoth_* = 16.9 mN m^−1^. The rather high *y* exponent value (=14.7) points to the complex interfacial architecture with significant spatial segregation of surface-active components of differentiated surface activity [4], as evidenced for all the studied mouthrinses (ranging from 12.9 to 15.5; see Table 3). The surface entropy *S_S_* determined from the *γ_LV_(T)* relation is unequivocally related to the molecular arrangements at the interface mediated by surface active components. Surface entropy decreases with an increase in surface adsorption *Γ_LV_* ↑ [16]. These two quantities, *y* and *S_S_*, are highly inversely correlated to each other (R = −0.986) and can be expressed with a linear fit function, i.e., *y* = B*S_S_* + A, where A = 18.43 (±0.40), B = −24.67 (±2.34).

### 4.4. Interfacial Viscoelasticity Modules

In the framework of a model for dilatational viscoelasticity adapted to the step-wise deformation mode, the real and imaginary parts of *E* (see Equations (4a) and (4b)) can be obtained from the following relations [17]:

(10a) $$E_{d}=E_{0}\frac{1+\Omega }{1+2\Omega +2\Omega^{2}}$$

(10b) $$E_{i}=E_{0}\frac{\Omega }{1+2\Omega +2\Omega^{2}}$$

where $E_{0}=\frac{\Pi_{0}-\Pi_{\infty }}{\Delta A/A_{0}\;}$; $\Omega =\sqrt{\frac{\Delta t}{\tau_{i}}}$, $\frac{\Delta t}{\tau_{i}}=ln\frac{\Pi_{\infty }-\Pi_{t}}{\Pi_{\infty }-\Pi_{0}}$; $\tan\varphi =\frac{\Omega }{1+\Omega }$; and *Π*_0_, *Π∞*, and *Π_t_*, are the surface pressures derived from *Π*(*t*) dependence at time *t* = 0, at the equilibrium (t = ∞) and at a specific time *t*. The relaxation times are given by *τ_i_*.

**Figure 4:**
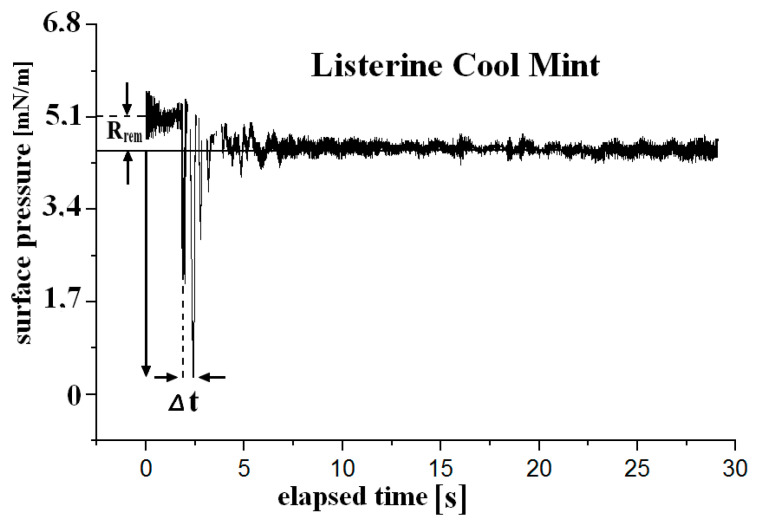
Results of stress–relaxation experiments: *Π(t)* decay dependence of surface pressure vs. time after a rapid step-wise (Δ*t* = 0.50 s) relative area compression (Δ*A/A*_0_ = 0.19) of the *A/L* interface for Listerine Cool Mint at *C = C*_0_, *T* = 22 °C, $t_{obs}$ = 2.62 s, *τ*_1_ = 0.63 and *τ*_2_ = 0.65 s, *|E|* = 10.3 mN m^−1^, *E_isoth_* = 1.3 mN m^−1^, *E_i_/E_d_* = 0.47, *De* = 0.24, and *R_rem_* = 0.7 mN m^−1^.

The exemplary decay *Π(t)* curve in Figure 4 can be approximated using a double exponential relation, *Π*(t) = *Π*_1_ exp(−t/τ_1_)+ *Π*_2_ exp(−t/τ_2_), according to a two-step transition process that likely takes place at the interface with relaxation times *τ*_1_ = 0.63 s and *τ*_2_ = 0.65 s. For the remaining liquids, τs were in rather narrow ranges, *τ_1_* (0.41–0.89) and *τ_2_* (0.64–3.13) s. Thus, $t_{obs}$ in this stress–relaxation experiment varied from 1.04 to 5.25 s, which corresponded to *De* (0.13–0.47). The film’s response to deformation with much longer times (Eurodont—3.1 s) indicates a greater complexity of the film structure and the occurrence of conformational processes with a longer time scale, similar to that exhibited by protein layers [18].

The interfaces exhibited an apparently viscoelastic behavior with the significant contribution of the imaginary part *E_i_* in the total *|E|*, as collected in Table 4. *E_i_/|E|* = 0.38–0.43 accompanied with large values of the loss angle φ (22.5–25.7°) despite being a kind of liquid [19]. The residual surface pressure *R_rem_* (0.1–1.4 mN m^−1^) is a quantitative measure of irreversible molecular reconfiguration processes in the layer as a result of the stress introduced, i.e., the film surface pressure never comes back to its initial value (see *R_rem_* at Figure 4). Dynamic dilatational elasticity modulus *|E|* was higher by a factor of 2.4–7.9 in reference to the steady-equilibrium state *E_isoth_* parameter. As a consequence, the effective surface tension *γ_eff_* differs from the static *γ_LV_*. Differences in *|E|* Δ*A/A*_0_ were achieved for Ortho Salvia Dental (3.1–14.3), Dentalux (+alc.) (4.8), Dentalux (alc. free) (3.1), Listerine Cool Mint (0.8–1.9), and Eurodont (4.2) mN m^−1^. Finally, it can be noticed that |E| increases with a decrease in τ leading to *γ_eff_* ↓, and consequently, since *CAH* ↑, *W_A_* is lowered for liquids forming more compact films (of higher elasticities).

The proportionality between *|E|* and Δ*A/A_0_* denotes the linear behavior range of the viscoelasticity of the system, as illustrated in Figure 8 of [20], occurring for homogeneous and isotropic interfacial systems. Such a linear behavior range of the interfacial system response to surface deformation (Δ*A/A*_0_), i.e., Δ*Π = |E|* Δ*A/A*_0_, can be determined from the *|E|* as a function of Δ*A/A*_0_ dependence. Considering four measuring runs data for Ortho Salvia Dental in Table 4, a monotonic increase in *|E|*, which means a deepening of the nonlinearity of the system, is evidenced for Δ*A/A*_0_ > 0.12.

## 5. Conclusions

The parameters of the adhesive properties of oral hygiene fluids in contact with a polymer substrate obtained in previous studies assumed a static, equilibrium state of the liquid–solid interface [1,2]. This allowed us to estimate the rate of expansion of the liquid contact line along the substrate under steady-state flow conditions. The penetration coefficient (PC) quantifies the liquid penetration process into capillaries [1,2]). For the metallic substrata studied here, PC values are similar, in the range of 5.2 (Listerine Cool Mint) to 7.8 (Ortho Salvia Dental) · 10^−2^ m s^−1^.

Under these conditions, the flow is characterized by Ca numbers (1.8–2.7) · 10^−3^ (in this work) and Bond and Weber numbers << 1. Nevertheless, even for such small PC values, the dynamic contact angle *θ_d_* (40.4–48.4°) is apparently higher than *θ_Y_* (20.7–33.3°), which leads to significant changes in the wetting parameters. Under conditions of intensive, stimulated rinsing of the oral cavity with hygiene fluids, PC values (data hardly available) can be many times higher. Furthermore, the viscoelastic properties of the surface rheology of the *A/L* surface lead to a significant change in the second parameter mediating the wettability of the solid substratum, i.e., surface tension from *γ_LV_* to *γ_eff_*, which depends on the complex dilatational viscoelasticity modulus *|E|*.

Values of the parameter *y* (=12.9–15.5) obtained using the 2D scaling theory of polymer films for the *L/V* interface layers of mouthrinse fluids revealed their significant molecular architecture heterogeneity in the molecular distribution of individual components depending on their own surface activity, which was strongly negatively correlated to the thermodynamic surface entropy *S_S_* (higher *S_S_* values point to a less complex surface layer structure). The values of the partition coefficient *a* = *Γ_SL_/Γ_LV_* and the variation of the adhesional stress as a function of *γ_LV_* proved the hydrophilic surface interactions at the *L/S* surface, taking place in the adsorption process opposite the PMMA surface case.

Moreover, at the contact line of two types of materials, in a composite prosthesis of polymer + metal with different hydrophobicities—*γ_SV_* (metal) > *γ_SV_* (polymer-PMMA, for example)—there will be a surface energy gradient d*γ_SV_*/d*x*, where d*x* is the length of the transition region in the direction along the composite surface, here, the wire diameter. For example, for Ortho Salvia Dental fluid (*γ_LV_* = 39.9 mN m^−1^) and surface free energies *γ_SV_* (metal) = 34.3 and *γ_SV_* (PMMA − polymer) = 22.6 mJ m^−2^, there will be a shear stress σ = *γ_LV_*[cos *θ_A_* (metal) − cos *θ_A_* (polymer)]/2r = 5.21 N m^−2^, which will cause the fluid to flow from the polymer-occupied area to the metal one (due to the Marangoni effect) [10].

The fluid velocity *u* in this phenomenon results from the equilibrium condition of shear stresses σ (Marangoni) and that of the viscous nature at the interface, which additionally depends on the dynamic viscosity of the liquid μ and the liquid layer thickness z, i.e., σ = d*γ_SV_*/d*x* = −μ d*u*/d*z*.

To sum up, determining the actual, trustful adhesive and wetting properties of medical hygiene fluids in contact with oral cavity solid substrata, which affects the effectiveness of the cleaning treatment, requires several additional parameters from the fields of surface rheology kinetics and thermodynamics to be accounted for. The evidenced particular variability of the film visco-elasticity (|*E*| ↑, *τ* ↓) and wettability (*θ_A_* ↑, *CAH* ↑) parameters resulted in adhesion (*W_A_* ↓), effective surface tension (γ_eff_ < γ_LV_), and penetration speed (*PC* ↓) decreasing. In further studies, the viscoelastic film properties related to the bioadhesion and antibacterial efficiency resulting from long-term treatment, temperature variability, or particular chemical components selection would be of great value for manufacturers and orthodontists.

## Figures and Tables

**Table 1:** Adhesive and wettability parameters for mouthrinse–orthodontic metal wire systems at *T* = 22 °C, derived from *CAH* = (*θ_A_* − *θ_R_*) [deg], and liquid surface tension *γ_LV_* [mN m^−1^] data. Mean values and standard deviations (in brackets).

Liquid	*CAH* [deg]	*Π* [mN m^−1^]	*W_A_* [mJ m^−2^]	*γ_LV_cosθ_A_* [mN m^−1^]	γ_*SV*_ [mJ m^−2^]	$\gamma_{SV}^{d}$ [mJ m^−2^]	$\gamma_{SV}^{p}$ [mJ m^−2^]	*a* = *ΓSL/ΓLV*
Ortho Salvia Dental θ_A_ = 39.3 θ_R_ = 26.8 γ_LV_ = 39.9	12.5 (0.3)	4.7 (0.1)	70.8 (4.1)	30.9 (1.8)	34.3 (0.9)	31.4 (1.1)	2.9 (0.1)	+0.77 (0.06)
Dentalux + alcohol θ_A_ = 28.1 θ_R_ = 16.0 γ_LV_ = 31.7	12.1 (0.4)	2.5 (0.1)	59.7 (3.6)	27.9 (1.3)	29.2 (0.9)	28.1 (1.2)	1.2 (0.1)	+0.88 (0.06)
Dentalux (alc. free) θ_A_ = 26.4 θ_R_ = 13.0 γ_LV_ = 29.7	13.4 (0.5)	2.3 (0.1)	56.3 (3.2)	26.6 (1.3)	27.6 (0.8)	26.7 (1.1)	0.9 (0.1)	+0.89 (0.06)
Listerine Cool Mint θ_A_ = 34.9 θ_R_ = 23.7 γ_LV_ = 37.6	11.2 (0.2)	3.6 (0.1)	68.4 (4.6)	30.8 (1.2)	33.4 (1.2)	31.1 (1.3)	2.2 (0.2)	+0.82 (0.08)
Eurodont θ_A_ = 26.7 θ_R_ = 12.2 γ_LV_ = 33.7	14.5 (0.3)	2.8 (0.1)	63.8 (4.2)	30.1 (1.3)	31.2 (1.0)	30.2 (1.2)	1.0 (0.1)	+0.89 (0.07)

**Table 2:** Wettability parameters for Ortho Salvia Dental–orthodontic metal wire system (*T* = 22 °C) versus mouthrinse relative concentration i.e., *C/C*_0_; *C*_0_—original product concentration, *CMC* = 0.03*C*_0_. Data, for *C~CMC* are marked in bold. Mean values and standard deviations (in brackets).

C/C_0_	*θ_A_* [deg] (0.2°)	*θ_R_* [deg] (0.2°)	*γ_LV_* [mN m^−1^]	*Π* [mN m^−1^]	*W_A_* [mJ m^−2^]	$\gamma_{SV}$ [mJ m^−2^]	$\gamma_{SV}^{d}$ [mJ m^−2^]	$\gamma_{SV}^{p}$ [mJ m^−2^]	*γ_LV_cosθ_A_* [mN m^−1^]
1	39.3	26.8	39.9 (1.1)	4.7 (0.2)	70.8 (2.2)	34.3 (1.4)	31.4 (1.1)	2.9 (0.2)	30.9 (1.3)
0.5	42.6	25.9	40.0 (1.3)	5.2 (0.2)	70.9 (2.1)	33.8 (1.3)	30.6 (1.2)	3.1 (0.2)	29.7 (1.2)
0.25	44.2	25.3	40.2 (1.3)	7.5 (0.3)	69.0 (2.1)	32.7 (1.3)	29.6 (1.2)	3.1 (0.2)	28.8 (1.1)
0.125	45.3	24.8	41.1 (1.3)	8.4 (0.3)	70.0 (2.3)	33.0 (1.2)	29.8 (1.3)	3.2 (0.2)	28.9 (1.3)
0.0625	46.2	23.2	41.8 (1.3)	9.5 (0.3)	70.7 (2.4)	33.1 (1.4)	29.9 (1.3)	3.3 (0.2)	28.9 (1.3)
**0.0313**	**48.4**	**23.8**	**42.7 (1.3)**	**10.7 (0.4)**	**71.1 (2.4)**	**33.0 (1.4)**	**29.5 (1.2)**	**3.5 (0.3)**	**28.4 (1.3)**
0.0156	52.2	24.0	44.3 (1.3)	13.3 (0.4)	71.5 (2.5)	32.7 (1.4)	28.8 (1.2)	3.9 (0.3)	27.2 (1.2)
0.0078	56.1	24.3	45.8 (1.4)	16.2 (0.5)	71.3 (2.3)	32.0 (1.3)	27.8 (1.2)	4.3 (0.3)	25.5 (1.2)
0.0039	55.3	24.5	48.2 (1.3)	16.4 (0.5)	75.6 (2.2)	34.1 (1.3)	29.7 (1.3)	4.4 (0.4)	27.4 (1.3)
0.001953	58.2	24.7	49.3 (1.3)	18.8 (0.4)	75.3 (2.3)	33.5 (1.3)	28.7 (1.3)	4.7 (0.3)	25.9 (1.4)
0.000976	60.3	24.9	54.3 (1.3)	22.3 (0.4)	81.2 (2.4)	35.7 (1.4)	30.4 (1.3)	5.3 (0.3)	26.9 (1.4)
0.000488	61.7	25.2	67.2 (1.3)	28.9 (0.3)	99.1 (2.6)	43.2 (1.5)	36.5 (1.4)	6.7 (0.3)	31.9 (1.4)
0.000244	62.9	25.4	72.3 (1.4)	32.4 (0.4)	105.6 (2.8)	45.8 (1.6)	38.4 (1.4)	7.3 (0.3)	33.1 (1.4)

**Table 3:** Interfacial structure complexity exponent *y* for the studied mouthrinses (at *C* = *C*_0_; *T* = 22 °C), derived from the *E_isoth_(Π)* dependence of the surface isotherm *(Π -A)_T_* origin, and surface entropy *Ss* (=−d*γ_LV_*/d*T*); data from Table 3 of [1].

Liquid	*y*	*S_S_* [mN m^−1^ K^−1^]
Ortho Salvia Dental	15.3 (0.6)	0.13 (0.02)
Dentalux (+Alc)	13.1 (0.4)	0.21 (0.04)
Dentalux (Alc. Free)	12.9 (0.3)	0.23 (0.04)
Listerine Cool Mint	14.7 (0.3)	0.14 (0.02)
Eurodont	15.5 (0.7)	0.12 (0.04)

**Table 4:** Rheokinetic parameters of surface dilatational viscoelasticity for mouthrinse liquids at *T* = 22 °C. Mean values and standard deviations or experimental uncertainties (in brackets).

Liquid	Δ*A/A*_0_·10^2^ (<3%)		Δ*t* [s]·10^2^ (1 ms)		$t_{obs}$ [s]		*τ*_1_ [s]		*τ*_2_ [s]	
Ortho Salvia Dental	12		46		3.83 (0.07)		0.74 (0.03)		0.92 (0.07)	
	8		42		5.25 (0.11)		0.81 (0.05)		0.87 (0.06)	
	8		36		4.50 (0.09)		0.59 (0.02)		0.85 (0.09)	
	27		28		1.04 (0.02)		0.41 (0.02)		0.64 (0.05)	
Dentalux + alcohol	12		61		5.08 (0.10)		0.71 (0.04)		0.79 (0.08)	
Dentalux (alc. free)	12		36		3.10 (0.07)		0.72 (0.03)		0.92 (0.11)	
Listerine Cool Mint	19 11		50 66		2.62 (0.05) 6.13 (0.13)		0.63 (0.02) 0.78 (0.04)		0.65 (0.05) 0.72 (0.07)	
Eurodont	24		45		1.87 (0.03)		0.89 (0.04)		3.13 (0.62)	
**Liquid**	***E_d_*** **[mN m^−1^]**	***E_i_*** **[mN m^−1^]**		***|E|*** **[mN m^−1^]**		***φ*** **[^o^]**		***R_rem_*** **[mN m^−1^]**		***E_isoth_*** **[mN m^−1^]**
Ortho Salvia Dental	24.1 (0.9)	10.6 (0.3)		26.3 (1.5)		23.7 (0.3)		0.2 (>0.1)		3.3 (0.2)
	37.0 (1.3)	15.5 (0.4)		40.1 (1.9)		22.7 (0.3)		0.2 (>0.1)		
	46.2 (1.8)	20.2 (0.5)		50.4 (2.5)		23.6 (0.3)		0.3 (>0.1)		
	48.4 (1.6)	21.9 (0.5)		53.1 (2.5)		24.3 (0.2)		0.4 (>0.1)		
Dentalux + alcohol	36.4 (1.4)	17.5 (0.4)		40.4 (2.3)		25.7 (0.3)		0.1 (>0.1)		5.6 (0.3)
Dentalux (alc. free)	24.1 (1.0)	10.6 (0.3)		26.3 (1.8)		23.7 (0.2)		0.3 (>0.1)		3.4 (0.2)
Listerine Cool Mint	9.3 (0.6) 6.7 (0.5)	4.4 (0.1) 3.2 (0.1)		10.3 (1.0) 7.4 (0.8)		25.2 (0.3) 25.6 (0.3)		0.7 (>0.1) 0.3 (>0.1)		1.3 (0.1)
Eurodont	16.1 (1.1)	6.7 (0.2)		17.4 (1.6)		22.5 (0.2)		1.4 (>0.1)		7.3 (0.4)

## Data Availability

The original contributions presented in this study are included in the article. Further inquiries can be directed to the corresponding author.
